# Monitoring tropical forest carbon stocks and emissions using Planet satellite data

**DOI:** 10.1038/s41598-019-54386-6

**Published:** 2019-11-28

**Authors:** Ovidiu Csillik, Pramukta Kumar, Joseph Mascaro, Tara O’Shea, Gregory P. Asner

**Affiliations:** 1 Center for Global Discovery and Conservation Science, Arizona State University, Tempe, AZ USA; 2 Planet Labs Inc, San Francisco, CA USA

**Keywords:** Forest ecology, Tropical ecology

## Abstract

Tropical forests are crucial for mitigating climate change, but many forests continue to be driven from carbon sinks to sources through human activities. To support more sustainable forest uses, we need to measure and monitor carbon stocks and emissions at high spatial and temporal resolution. We developed the first large-scale very high-resolution map of aboveground carbon stocks and emissions for the country of Peru by combining 6.7 million hectares of airborne LiDAR measurements of top-of-canopy height with thousands of Planet Dove satellite images into a random forest machine learning regression workflow, obtaining an R^2^ of 0.70 and RMSE of 25.38 Mg C ha^−1^ for the nationwide estimation of aboveground carbon density (ACD). The diverse ecosystems of Peru harbor 6.928 Pg C, of which only 2.9 Pg C are found in protected areas or their buffers. We found significant carbon emissions between 2012 and 2017 in areas aggressively affected by oil palm and cacao plantations, agricultural and urban expansions or illegal gold mining. Creating such a cost-effective and spatially explicit indicators of aboveground carbon stocks and emissions for tropical countries will serve as a transformative tool to quantify the climate change mitigation services that forests provide.

## Introduction

Mitigating the effects of climate change is a critical societal objective now and in the forthcoming decades. Tropical countries contribute to carbon emissions mainly through deforestation and forest degradation, which accounts for approximately 10% of the world’s annual total carbon emissions^1^. National and international initiatives such as REDD+^2^ are dedicated to reducing carbon emissions from deforestation and forest degradation. To achieve this objective, each nation’s carbon emissions resulting from deforestation and forest degradation need to be quantified and tracked over time^3^. At such large geographic scales, a precise, cost-effective and high-resolution means to monitor changes in aboveground carbon stocks is needed.

Traditionally, forest carbon stocks have been estimated using field plot networks by correlating tree structural characteristics (diameter, height and wood density) to aboveground carbon density (ACD) using allometric equations^4^. While this approach may be suitable for local areas, airborne LiDAR (light detection and ranging) has proven useful in extending ACD mapping estimation outside of necessarily limited field plot inventory networks^5^. LiDAR measurements provide detailed three-dimensional information of the forest canopy height and structure, and it was shown that LiDAR- and field-based estimation of carbon stocks have an agreement of ~90% when calibrated at 1-ha spatial resolution^5,6^. At this resolution, the 10% difference is smaller than the error usually encountered in field-based estimation of ACD^7^. LiDAR can be used, therefore, to extend the mapping of carbon stocks to areas larger than field-based estimates can provide, and has been used successfully for ACD or biomass mapping in tropical forested regions of South America^8^, Central America^6^, Africa^9–11^, Asia^12^ or oceanic islands^13^. Although LiDAR can extend the analysis to larger areas, it also reaches a geographic limit determined by costs and logistical aspects associated with the use of aircraft^14^. To overcome this, LiDAR is often used with optical remote sensing data of various spectral and spatial properties to scale from airborne to full-coverage, satellite-based areas^8,15–17^.

Combining satellite images and other geospatial datasets with airborne LiDAR has become a common approach to map forest ACD across regions that lack LiDAR measurements^18,19^. Different spatial resolutions of satellite images have been used, from low and medium to high and very high spatial resolution. Baccini and Asner^15^ made use of Moderate Resolution Imaging Spectroradiometer (MODIS) images in combination with airborne LiDAR to generate pantropical ACD maps of greater details and improved accuracy. Asner *et al*.^8^ combined Landsat-derived metrics with Shuttle Radar Topography Mission (SRTM) elevation variables to calibrate an ACD model using airborne LiDAR data for the entire country of Peru. Using canopy texture metrics derived from high resolution Geoeye-1 and Quickbird images, Bastin *et al*.^20^ mapped aboveground biomass (AGB) for very heterogeneous African forest types. Hojas Gascon *et al*.^21^ used RapidEye (5 m) optical images to reduce the efforts in collecting national field data for estimating AGB over Tanzania. Hirata *et al*.^22^ developed an object-based approach to map the AGB in tropical forests of Cambodia using a combination of airborne LiDAR with QuickBird images. While medium and lower resolution satellite images have global coverage and are usually free of charge, the usage of high and very-high resolution satellite images in estimating ACD is limited to smaller study areas, due to their costs and availability^21^.

This spatial limitation of very-high resolution satellite image availability has recently changed via the rapid emergence and availability of Planet data streams^23^. Planet Labs Inc. operates the largest fleet of Earth imaging satellites, with around 180 “Dove” satellites currently in orbit and imaging the entire Earth, every day. Dove imagery have been successfully used to map coral reefs and seagrass ecosystems^24^, benthic habitats^25^, agricultural environments^26–28^, and digital elevation models have been successfully generated from multi-view Dove imagery^29^. Imaging the Earth daily using four spectral bands (blue, green, red and near-infrared) at a resolution of 3.7 m will contribute in overcoming an important issue when using satellite images in estimating ACD, namely the cloud coverage of tropical regions. To our knowledge, Planet Dove images have not been used yet to map aboveground carbon stocks for tropical forests at large extents.

Estimating forest properties, like ACD or tree canopy height, from optical images is accomplished using various machine learning regression models^30–33^. Of these, the Random Forest (RF) algorithm^34^ has proven to be superior to traditional techniques for carbon mapping applications, such as regionally stratified sampling and upscaling^35^. Part of this success is because RF is non-parametric, robust to a high number of input variables and insensitive to data skew^36^. RF regression techniques have been intensively used to map carbon stocks^8,35^, biomass^21,31^ or tree canopy height^37,38^, for a broad range of spatial resolutions of satellite images. When dealing with high and very high resolution images, besides spectral reflectance, band ratios and indices derived from these, common features used in an RF regression are related to image texture, which detects forest canopy structural heterogeneity and ultimately predicts variations in ACD^20^. Two very popular textural measures used as a remotely sensed vegetation structure feature are the grey-level co-occurrence matrix (GLCM) texture^39–42^ and Fourier transform textural ordination (FOTO)^20,43–45^. Although textural features have been applied to various sensors such as IKONOS-2^42^, Cartosat-1a^46^, SPOT-5^41^, QuickBird^47,48^, WorldView-2^49^, or RapidEye^21^, it was not tested on how it performs using Planet Dove images for large scale mapping of ACD.

Building on the previous work of Asner *et al*.^8^, who developed a country-wide ACD modeling framework for Peru, our study aims to estimate ACD at an unprecedented level of detail for the entire country of Peru by making use of Planet Dove spectral and textural features in combination with airborne LiDAR, integrated into an efficient RF-machine learning regression framework. Furthermore, we compared our ACD results with those from Asner *et al*.^8^ to calculate the carbon emissions and sinks for the period between 2012 and 2017 and show examples of significant carbon changes through time led by oil palm and cacao plantations, urban expansions and gold mining. Achieving high resolution ACD estimates at 1-ha resolution will greatly contribute to a rapid and cost-effective monitoring system of carbon emissions for REDD + initiatives.

## Results

### Top-of-canopy height (TCH) estimation and uncertainties

**Figure 1:**
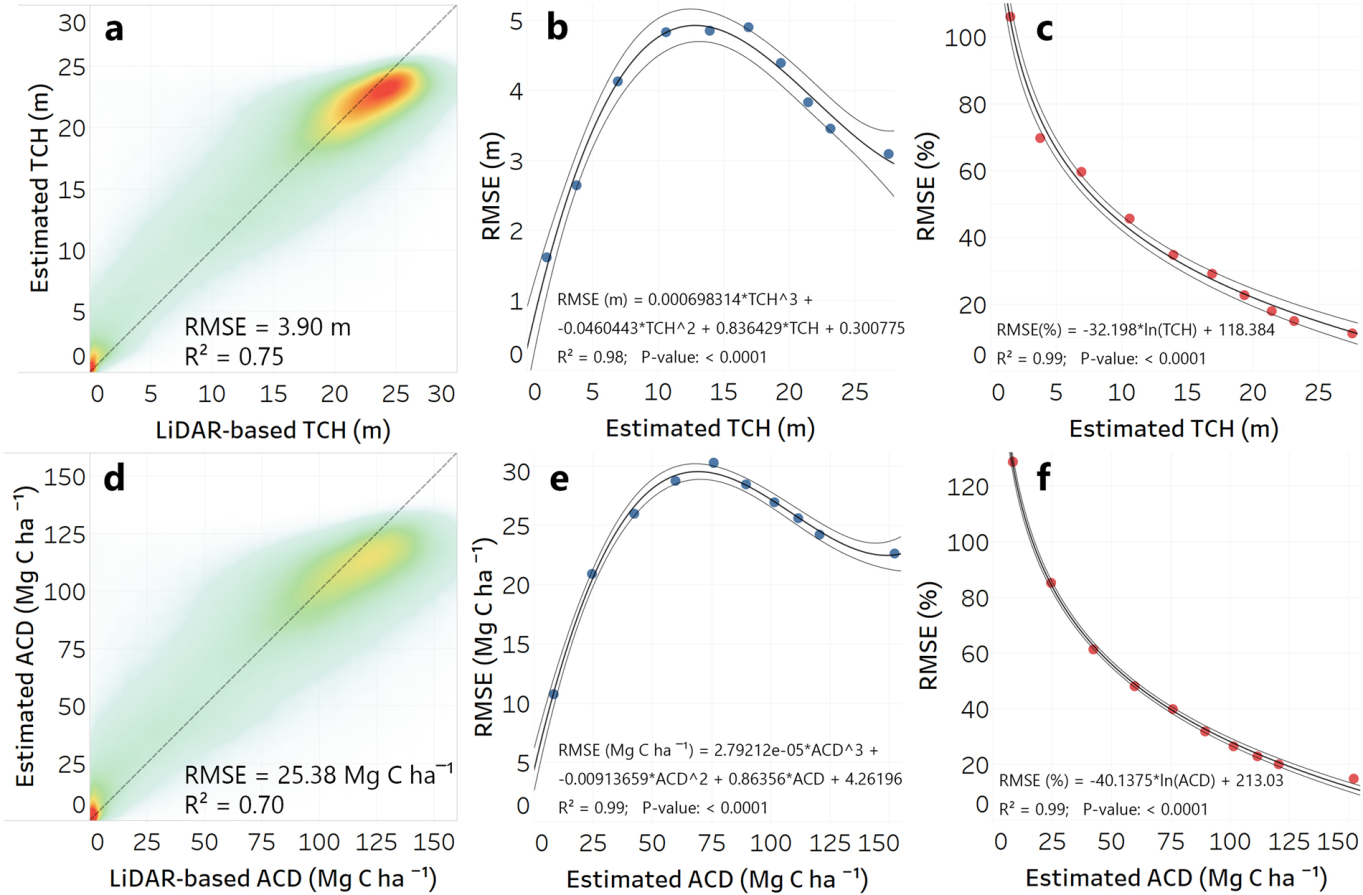
Density scatter plot depicting the relation between RF-estimated TCH and LiDAR-measured TCH, using more than 1.3 mil 1-ha validation samples (**a**). Uncertainty of RF-estimated TCH expressed as root mean squared error (RMSE, in m) of RF-estimated TCH, with a polynomial function fitted (**b**). Decline in uncertainty (in %) of RF-estimated TCH with increasing TCH values, with a natural logarithm function fitted (**c**). Density scatter plot depicting the relation between RF-estimated ACD and LiDAR-measured ACD, using more than 1.3 mil 1-ha validation samples (**d**). Uncertainty of RF-estimated ACD expressed as root mean squared error (RMSE, in Mg C ha^−1^) of RF-estimated ACD, with a polynomial function fitted (**e**). Decline in uncertainty (in %) of RF-estimated ACD with increasing ACD values, with a natural logarithm function fitted (**f**).

The TCH estimates yielded an R^2^ of 0.75 and a root mean square error (RMSE) of 3.90 m when compared with LiDAR-based TCH validation samples (Fig. 1a). Grouping the RMSE results into 10 bins with a fitted function led to estimated RMSE of less than 4 m for RF-estimated TCH of less than 7 m. TCH values of 7 to 21 m had an estimated RMSE in the form of negative parabola (opens downwards) with values from 4 to 4.9 m with the peak around the value of 13 m for estimated TCH (Fig. 1b). Transforming the RMSE values into percentage RMSE of estimated TCH depicted a decline in uncertainty of RF-estimated TCH with increasing TCH values (Fig. 1c). This decline is desirable in the context of ACD mapping, since a vast amount of carbon stocks are stored in trees with heights of 15 m or more. Estimated TCH of 8 m or less had a percent RMSE of more than 50%. This error decreased up to 30% for TCH of 8 to 15 m. Estimated TCH of 15–21 m had 30% to 20% RMSE, while TCH values higher than 25 m had less than 15% RMSE of the estimated values. These values for the high trees were similar with the errors encountered during field measurement of TCH.

### ACD estimation and uncertainties

Transforming the RF-estimated TCH into ACD resulted in a nationwide ACD model with R^2^ of 0.70 and RMSE of 25.38 Mg C ha^−1^ when compared against the 1.3 mil hectares of LiDAR transformed into ACD (hereafter, LiDAR-derived ACD) (Fig. 1d). This was the first source of error in estimating the uncertainty of our ACD model. Again, grouping the RMSE into 10 natural breaks bins and fitting a polynomial function led to estimated RMSE values of less than 26 Mg C ha^−1^ for estimated ACD values of less than 40 Mg C ha^−1^. RMSE values between 26 and 30 Mg C ha^−1^ characterized ACD values of 40 to 110 Mg C ha^−1^. For values higher than 110 Mg C ha^−1^ the RMSE decreased while ACD increased, up to 22.5 Mg C ha^−1^ RMSE for 150 Mg C ha^−1^ estimated ACD (Fig. 1e). In terms of percent RMSE of ACD, there was a 50% or more RMSE for estimated ACD values of 60 Mg C ha^−1^ or less, 50 to 30% RMSE for ACD of 60 to 100 Mg C ha^−1^ (Fig. 1f). ACD values of 100 to 120 Mg C ha^−1^ had 30 to 20% RMSE, while estimated values higher than 120 Mg C ha^−1^ had less than 20% RMSE of its values.

**Figure 2:**
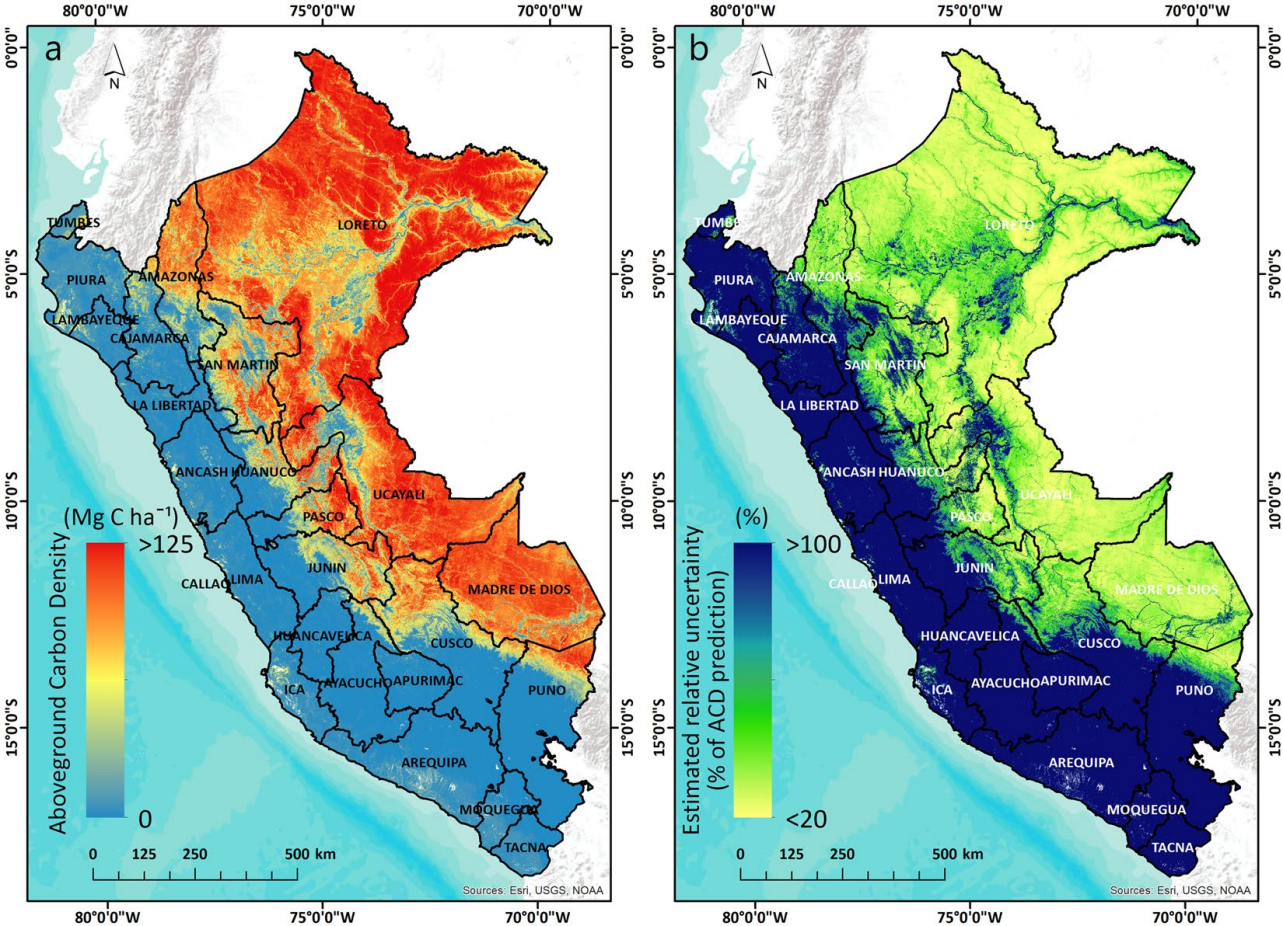
The high-resolution ACD map of Peru at 1-ha resolution expressed in Mg C ha^−1^ using Planet Dove satellite imagery (**a**). Estimated relative uncertainty expressed as a percentage of estimated ACD (**b**). The regions of Peru are shown in black outlines with their associated names.

To better estimate uncertainty in our ACD map, we also considered the second type of error attributable to calibrating the LiDAR-measured TCH to field-measured ACD (Eq. (1)). The mean error of LiDAR-based estimates of ACD had previously been reported at 11.6%^8^. Combining the two sources of error (Eq. (2)) resulted in a nationwide map of estimated relative uncertainty, expressed as a percentage of estimated ACD for every hectare (Fig. 2).

### High-resolution map of ACD and regional statistics

**Figure 3:**
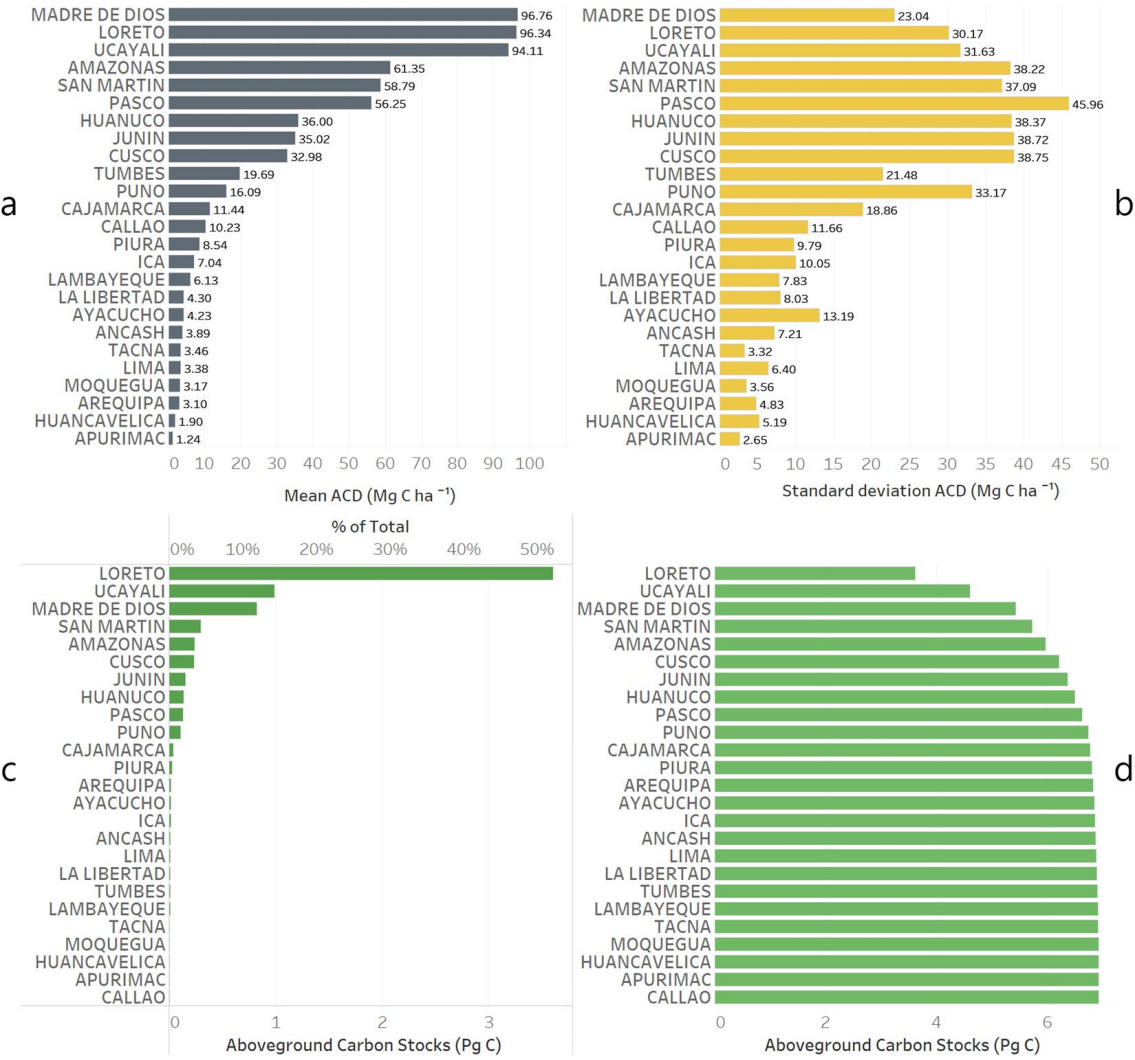
First level administrative subdivisions of Peru (regions) and their statistics of ACD, mean (**a**) and standard deviation (**b**). The regions are ordered by the mean ACD (Mg C ha^−1^). The same regions of Peru ordered by the total amount of aboveground carbon (in Pg C and % of total) and the cumulative graph are shown in (**c**,**d**), respectively.

The high-resolution map of Peru’s ACD revealed a wide range of ACD values, from <1 Mg C ha^−1^ in the western dry deserted areas to 150 Mg C ha^−1^ in the highest biomass lowland Amazonian forest in northeastern part of the country (Fig. 2). The total aboveground carbon stock estimated for Peru is 6.928 Pg with a country-scale uncertainty <1%, a value similar to the one obtained by Asner *et al*.^8^ for Peru, of 6.922 Pg. The estimated ACD ranged between 0 and 152.3 Mg C ha^−1^, with a mean of 53.91 Mg C ha^−1^, and standard deviation of 49.07 Mg C ha^−1^. The diversity of ecosystems in Peru drove the spatial arrangement of aboveground carbon stocks that is highly dependent on elevation, geological substrate, soil fertility, hydrological characteristics and climate^8,50^. Three legal jurisdictions in Peru store more than 78% (5.424 Pg) of country’s total aboveground carbon stocks, with Loreto sheltering 3.613 Pg (52.1%), Ucayali 0.99 Pg (14.2%), and Madre de Dios with 0.82 Pg (11.8%) (Fig. 3). The other 22 regions combined harbor 1.5 Pg C, with Western Amazonian regions of San Martin (0.3 Pg), Amazonas (0.24 Pg) and Cusco (0.23 Pg) having more than 0.2 Pg C.

Mean ACD values reached a maximum of 96.7 Mg C ha^−1^ in Madre de Dios, 96.3 Mg C ha^−1^ in Loreto and 94.1 Mg C ha^−1^ in Ucayali (Fig. 3). The highest standard deviations in ACD were found in regions that transition between high-biomass tropical forests to the eastern slopes of the Andes, like Pasco, Cusco, Junin, Huanuco, Amazonas, San Martin, and Puno (Fig. 3).

**Figure 4:**
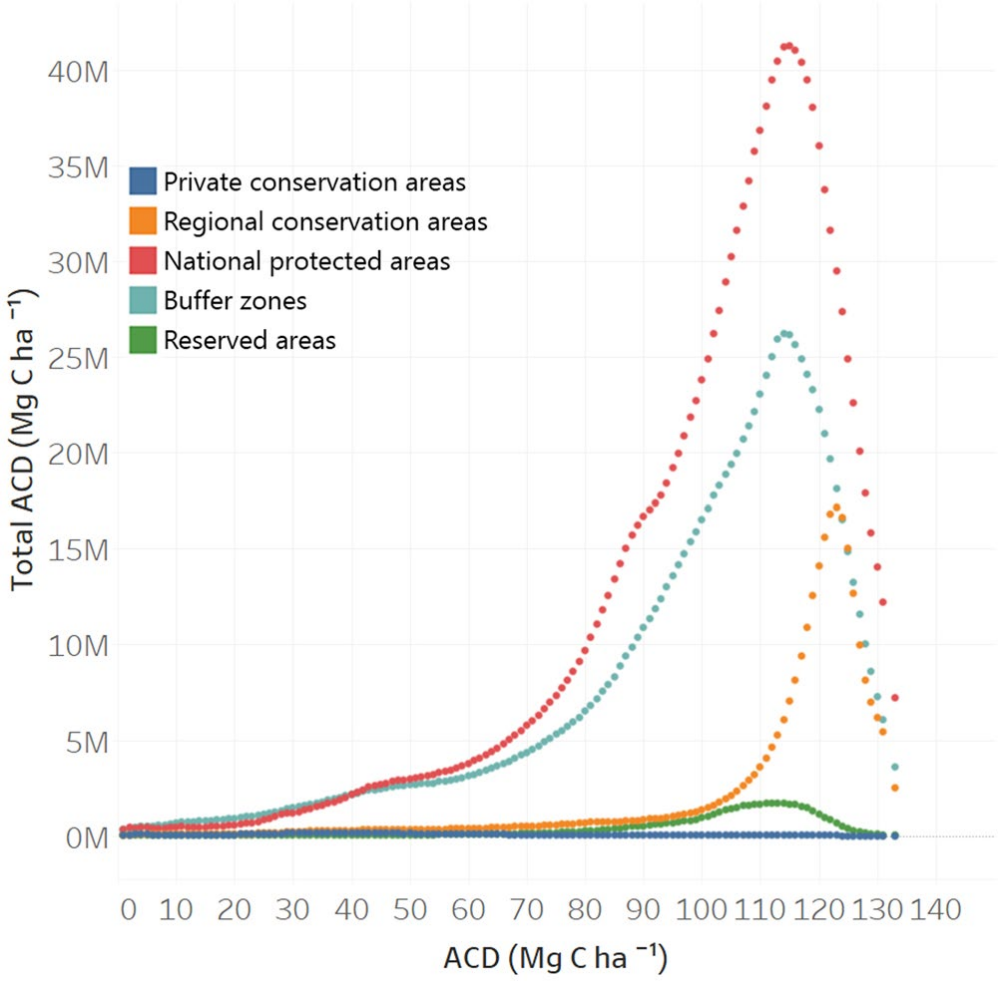
The total aboveground carbon stocks (Mg C ha^−1^) found in five types of protected regions across Peru.

From the total aboveground carbon stock of 6.928 Pg C estimated for Peru, 2.90 Pg C were found in five types of protected areas, private and regional conservation areas, national protected areas, reserved areas, and buffer zones (Fig. 4, Table 1). Of them, national protected areas with its buffer zones covered more than 32 mil hectares with more than 2.5 Pg C. The highest mean ACD was for regional conservation areas (92.37 Mg C ha^−1^), which had its three biggest conservation areas in high biomass forests of Loreto.

**Table 1:** Statistics of estimated ACD for multiple types of protected areas in Peru, totaling 2.90 Pg C.

Protection type	Area (mil ha)	Mean ACD (Mg C ha^−1^)	STD ACD (Mg C ha^−1^)	Total ACD (Pg C)
Private conservation areas	0.38	19.45	22.17	0.007
Regional conservation areas	3.09	92.37	43.99	0.285
National protected areas	18.44	83.35	39.36	1.537
Buffer zones	13.98	73.04	43.23	1.021
Reserved areas	0.63	78.87	43.26	0.049

### Carbon emissions between 2012 and 2017

**Figure 5:**
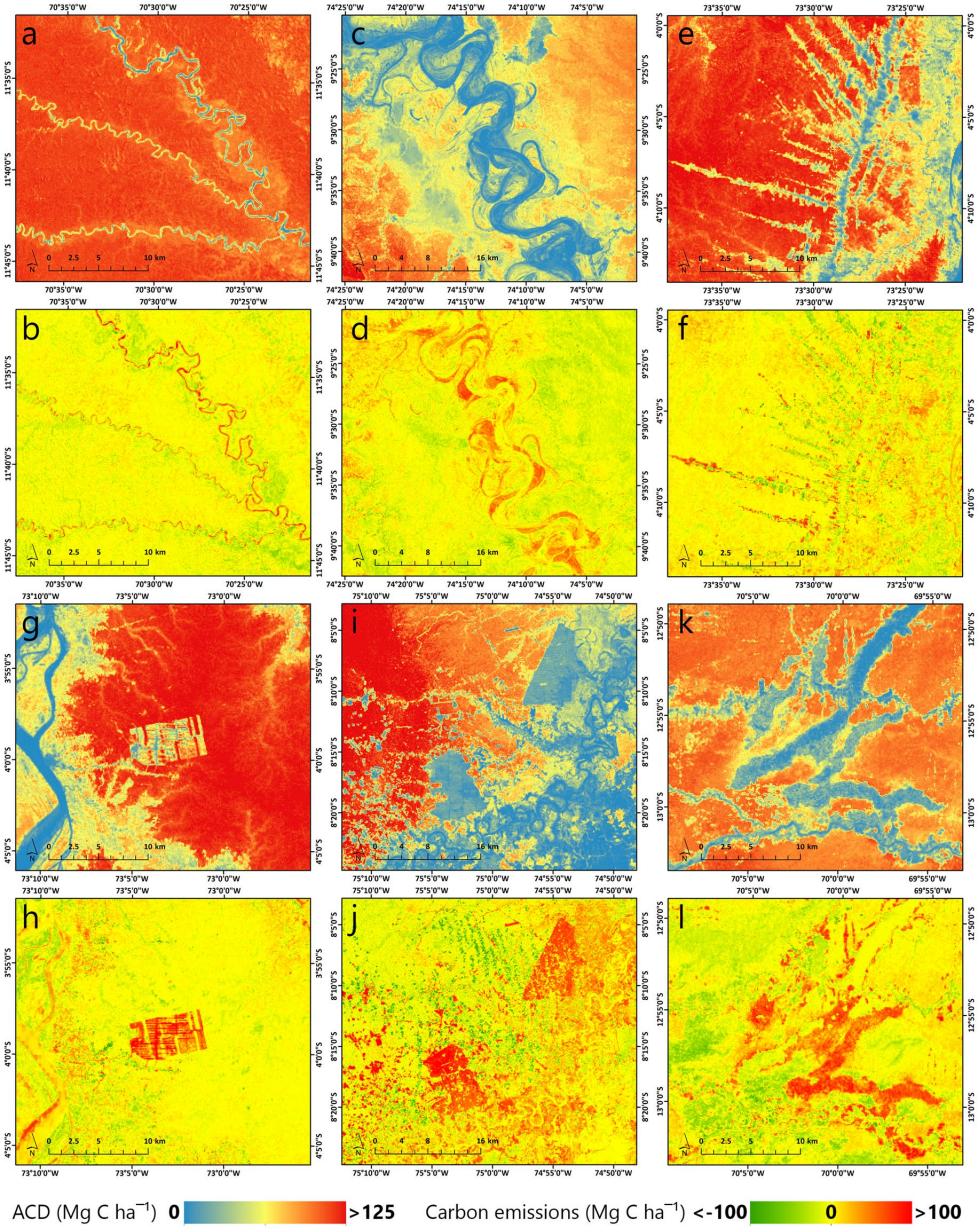
Examples of changes over time in ACD (Mg C ha^−1^), measured as the difference between our ACD estimates (2017) and the results from Asner *et al*.^8^ from 2012. Intact forests showed insignificant changes in ACD in southeastern Peru (**a,b**), while other sources of natural variation, like river meandering, created a buffer of carbon emission zones along its path (**c,d**). Human-dominated sources of carbon emissions were represented by deforestation along the Iquitos-Nauta road in Loreto (**e,f**), aggressive cocoa plantation expansion disturbing a high biomass forest near Iquitos (**g,h**), deforestation, forest degradation and two new large areas of oil palm plantation near the city of Pucallpa, Ucayali (**i,j**), and expansion of gold mining activities between Boca Colorado and Puerto Maldonado, in Madre de Dios (**k,l**).

We compared our ACD estimates with results from Asner *et al*.^8^ and developed a map of carbon stocks and emissions between 2012 and 2017 at 1-ha resolution. Using high-resolution Planet Dove images facilitated a high precision view of specific landscape elements, such as small rivers, as well as fine-scale disturbances and smooth transitions between vegetation types. Acknowledging the uncertainties of the two ACD maps and the artifacts that might result in comparing the two, some areas across Peru showed obvious signs of changed ACD that were identified by our analysis (Fig. 5). In Fig. 5, two examples of natural sources of variation are represented by vegetation dynamics with insignificant changes between the two time periods analyzed and river meandering that left a footprint of its hydrological evolution on the pattern of carbon emissions (Fig. 5a–d). Human activities impacted the distribution of ACD and are represented mainly by deforestation and forest disturbance along transportation routes, like in the case of Iquitos-Nauta road in Loreto (Fig. 5e,f), newly emerged large areas of cocoa plantation disrupting intact forests (e.g. near Iquitos, Fig. 5g,h) or oil palm plantations in areas that had already suffered major deforestation and land conversions (e.g. near Pucallpa, Fig. 5i,j). One of the major threats to carbon stocks is gold mining in the Madre de Dios region, with older mining areas expanding and new ones emerging at a rapid pace with irreversible impact over the environment (e.g. area between the cities of Boca Colorado and Puerto Maldonado, Fig. 5k,l).

**Figure 6:**
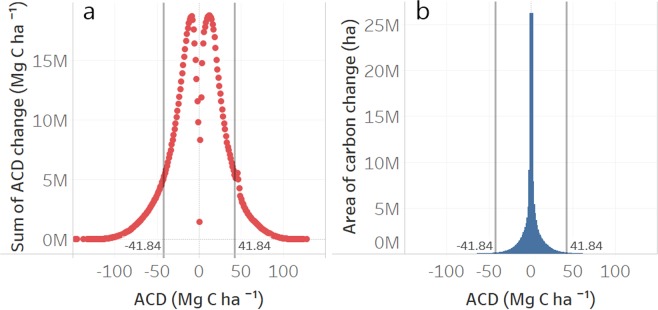
Statistics of carbon sink and emissions between 2012 and 2017, in terms of total amount of changed aboveground carbon stocks (**a**) and area occupied by the differences between the two maps (**b**). Vertical lines depict the combined RMSE errors of the two maps (41.81 Mg C ha^−1^).

Because the two maps were developed independently, we must be cautious in stating absolute values of carbon emissions. While the examples of carbon changes from Fig. 5 are unquestionable, we combined the RMSE errors of the two maps by computing the square root of the sum of the two, which resulted in a 41.84 Mg C ha^−1^ combined error. Using this value as a threshold, our present map of carbon changes estimated 0.08 Pg C as a carbon sink and 0.096 Pg C as carbon emissions between 2012 and 2017, while 0.02 Pg C fall between −41.81 and 41.84 Mg C ha^−1^ (Fig. 6). Regarding the area occupied by these differences, carbon emissions take 1.7 mil hectares, carbon sink 1.5 mil hectares, while the rest of 125 mil hectares are attributable to uncertain differences (Fig. 6).

## Discussion

We developed the first large-scale very high-resolution map of aboveground carbon stocks and change for the country of Peru by combining 6.7 million hectares of airborne LiDAR measurements of top-of-canopy height with thousands of Planet Dove satellite images into a random forest machine learning regression workflow, obtaining an R^2^ of 0.70 and RMSE of 25.38 Mg C ha^−1^ for the nationwide estimation of ACD.

Random forest regression was chosen due to its proven accuracy and ability to deal with large datasets^8,35^. Other studies have used an RF approach for estimating tree canopy height^37^, aboveground biomass^21^ or aboveground carbon stocks^8,35^. For example, our RMSE is close to what Mascaro *et al*.^35^ found while mapping tropical forest carbon in a 16 million hectare area in Western Amazon using RF with spatial context (RMSE of 26 Mg C ha^−1^) or similar to what Asner *et al*.^8^ found when estimating ACD at the country-scale of Peru (RMSE of 27.4 Mg C ha^−1^). We are aware that using local RF models fitted throughout Peru and then combining the results will make our approach less transferable to other similar study areas. However, it was previously shown that RF models trained using samples from all the study area can result in a universal model for canopy height estimation with R2 higher than 0.6 and RMSE lower than 6 m^38^. Our trained model can be applied to different areas with similar environmental conditions without the need for LiDAR data, or retrained for different forested ecosystems based on LiDAR and satellite imagery.

Creating a carbon estimation model using spectral and textural information from Planet Dove is changing the way how we approach a cost-effective, automated, timely, and spatially-explicit indicator of carbon stocks and emissions of tropical forests. Although we benefited from the high spatial and temporal resolution of the images, the Planet Dove mission is still in its early stages. One major issue is related to the cross-sensor inconsistencies^26^ that can lead to unwanted artifacts in the mosaic. These induced inconsistencies will propagate while using sensitive variables like the GLCM textures. Using more robust features extracted from Dove mosaic for TCH estimation will be one path for further investigations. We are also aware of the temporal differences between the datasets used, with LiDAR from 2011–2013 and Dove images from 2017. Using RF with multiple decision trees will neutralize the small number of LiDAR-sampled hectares that changed in this timeframe.

We estimated 6.928 Pg C stored as aboveground carbon stocks in Peru, a quantity similar to what Asner *et al*.^8^ mapped for the same country. While only 2.90 Pg C are found in protected areas or their buffer areas, many more high-carbon densities tropical forests are under threat by human activities, like oil palm plantation, gold mining or fossil fuel oil extraction. To achieve a net neutral carbon balance, we need not only to limit gross emissions of carbon, but also to transform more disturbed areas into carbon sinks. This is of critical importance in the context of severe and frequent droughts the Amazon forests have experienced, which diminishes the role of the Amazonian forests acting as carbon sinks^17^. For this, monitoring the carbon sequestrations and emissions through time will help for better actions and we showed in this study a first-time high-resolution Peru-wide estimates of carbon changes through time.

## Conclusion

The role of tropical forests in mitigating the effects of climate change is important and needs to be better understood. Here, we presented a large-scale mapping of aboveground carbon stocks at an unprecedented level of detail by combining high resolution Planet Dove images and airborne LiDAR into a cost-effective and robust random forest statistical approach. Furthermore, we showed a 1-ha resolution map of carbon emissions between 2012 and 2017, which can help improve understanding of land use changes and inform a sustainable pathway to economic development for tropical countries. Creating cost-effective, automated and spatially explicit indicators of aboveground carbon stocks for tropical countries has transformative potential for practically quantifying the climate change mitigation services forests provide, including within MRV (Measurement, Reporting, and Verification) systems for REDD + (Reducing emissions from deforestation and forest degradation) under the UNFCCC (United Nations Framework Convention on Climate Change).

## Data and Methods

### Study area

Our study area is the entire Republic of Peru, covering more than 128.5 million hectares. Forests of Peru are very high in biodiversity, with high mountainous Andean regions extending from northwest to southeast and tropical lowlands of the Amazonian Basin. These tropical forests are amongst the most biologically diverse regions in Amazonia^51^, with high tree species richness, reaching more than 300 species with diameter higher or equal with 10 cm in single hectares^52^. The highly diverse environmental and biological gradients, together with rapid land use changes specific for a developing economy, strongly influence the carbon storage throughout its ecosystems^8^.

### Airborne LiDAR data

**Figure 7:**
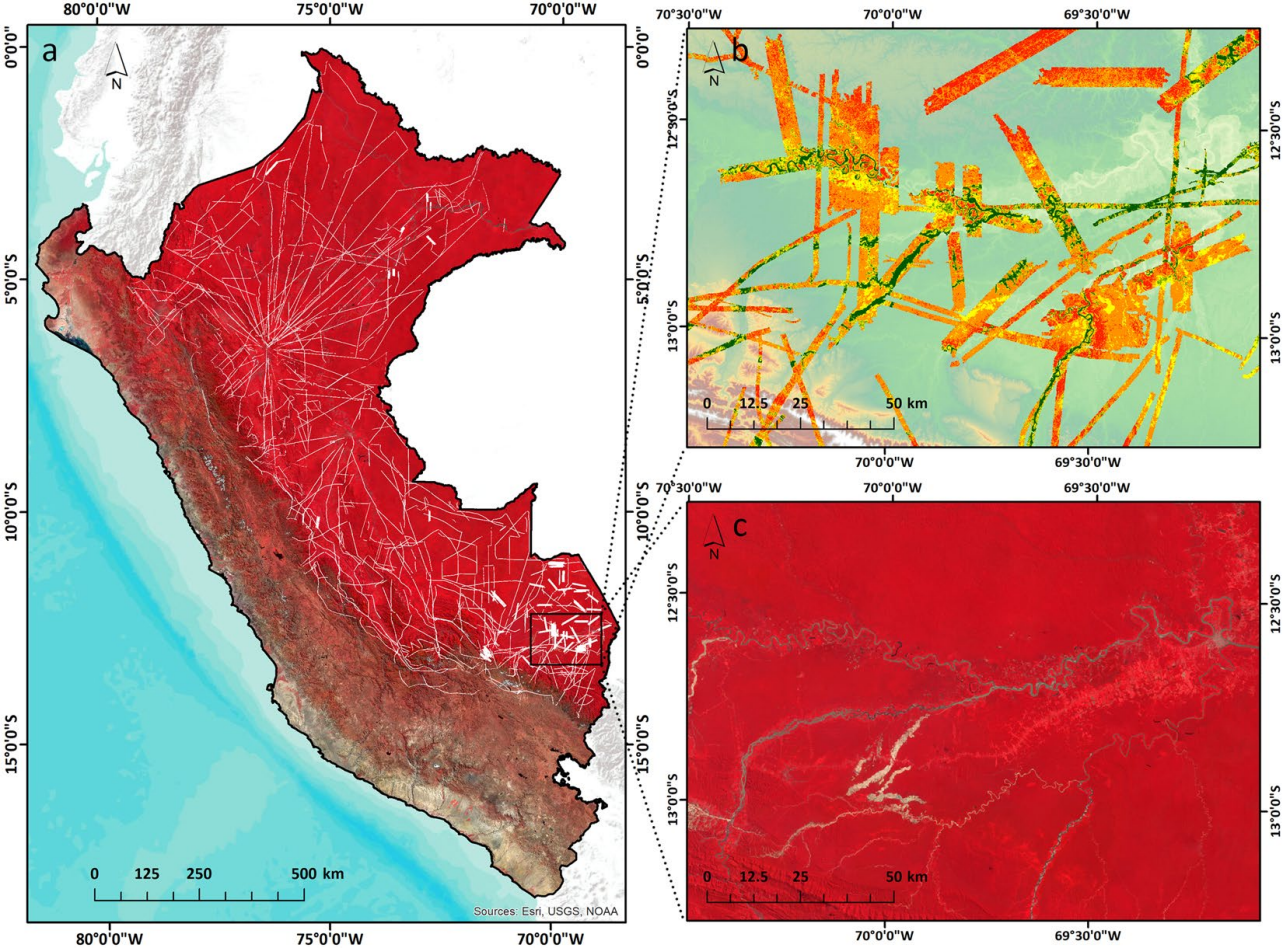
Flight paths and airborne LiDAR data acquired by the Global Airborne Observatory (white paths) overlapping a false color composite of Planet Dove mosaic (**a**). Two zoomed-in subsets of LiDAR (**b**) and Dove (**c**) are shown for a region that underwent forest disturbances through deforestation and gold mining. Both LiDAR and Dove mosaic are shown here at 1-ha resolution, as used in the random forest (RF) regression.

The airborne LiDAR data was acquired during 2011 and 2013 flight campaigns using the Global Airborne Observatory (GAO; formerly Carnegie Airborne Observatory)^53^ (Fig. 7). The GAO LiDAR is a dual-laser scanning waveform system capable of firing 500,000 laser shots per second, with up to four discrete returns per laser shot^53^. The aircraft was operated at an altitude averaging 2000 m above ground level with speeds of up to 150 knots. An average-on-the-ground LiDAR points spacing of 4 shots per square meter was achieved, with up to 8 shots per square meter in overlapping flight areas^8^. Thus, a 3D LiDAR point cloud with a resolution of 1.12 m was achieved, covering not only forested ecosystem, but also other less representative ecosystems, like grasslands, shrublands or savannas. The airborne LiDAR data were acquired on similar season as the PlanetScope imagery, thus minimizing possible differences due to seasonality of the vegetation.

From the 3D LiDAR point cloud, a digital terrain model (DTM) and a digital surface model (DSM) were constructed from the last and first returns, respectively. Subtracting DTM from DSM generated a top-of-canopy height (TCH) model at a spatial resolution of 1.1 m, covering 6,677,177 hectares throughout Peru (Fig. 7). This simple LiDAR metric, TCH, was shown to be effective in mapping ACD at 1-ha resolution^5^.

### Planet Dove satellite data

Planet Dove is a low-Earth orbital constellation comprised of approximately 180 CubeSat 3U form factor (10 cm by 10 cm by 30 cm) satellites operating in sun-synchronous orbit (475 km altitude). Dove satellites acquire images using four spectral bands, blue (455–515 nm), green (500–590 nm), red (590–670 nm) and near infrared (780–860 nm), at a ground sample distance at nadir of approximately 3–4 m and a positional accuracy of less than 10 m root mean square error (RMSE)^54^. To generate a seamless analysis-ready mosaic of Dove images for Peru, we combined analytic ortho scene products that previously had top of atmosphere radiance correction, surface reflectance atmospheric correction, have been normalized, resampled to 3.125 m and orthorectified using GCPs and fine DEMs to less than 10 m RMSE positional accuracy. We generated the mosaic using 73,466 Dove scenes from the dry season of 2017 (July 1 - September 30), which offered cloud-free pixels. The final cloud coverage of the mosaic was 0.8%, with isolated clouds over the Andes. (Fig. 7).

The normalized mosaic was created by applying a transformation to Dove surface reflectance (SR) data based on a linear fit of each scene’s data to co-registered Landsat data from a similar season. The transform is constrained to preserve values with a reflectance of 1.0, and to prefer darkening the scene to brightening it. The latter is because the MODIS AOT (Aerosol Optical Thickness) maps used for SR more often lead to underestimates of the atmospheric component than overestimates. As a result, the Dove SR scenes are frequently too bright, particularly in tropical areas. As a final step (optional, but recommended), a seamline removal algorithm was applied to make a long-wavelength adjustment to intensity near scene boundaries so that the values in adjacent scenes are similar, with values near a scene boundary changing more than values away from a scene boundary (i.e. gradient reconstruction). Note that the seamline removal is not blurring or feathering and does not affect the spatial resolution of the data. It does, however, shift the absolute values.

### Dove GLCM texture

Image texture is an important characteristic of every image that can help in identifying different objects or regions within an image by measuring the spatial arrangements of image tone intensities^39^. Image texture metrics have been widely used to measure vegetation structure characteristics^40,42^ and, furthermore, to estimate forest biomass and carbon stocks^55,56^. We used the gray level co-occurrence matrix (GLCM) texture^39^, which are one of the most popular texture metrics for remote sensing applications^48^. GLCM are second-order texture measures that compute how often pairs of pixels with similar brightness values (gray tones) appear in an image at a given spatial relationship. We computed eight GLCM texture measures, namely the mean, variance, homogeneity, contrast, dissimilarity, entropy, second moment and correlation using a window size of 3 × 3 pixels and a shift of 1 pixel, for every pixel in the image. We chose a small window size to capture the hard-to-detect detailed changes in the dense canopy structure of the tropical forests. We used 32 levels of gray and derived the texture in a single direction, of 135°. We used the Dove green band to compute the GLCM textures. All of these settings were decided after running tests on smaller areas to find the best solution in terms of accuracy and speed of computation.

### Random forest regression

Random Forest (RF) is an ensemble technique used for both classification and regression and have become widely popular for remote sensing applications due to the accuracy of its results^34,57,58^. RF uses multiple decision trees that are built independently using subsets of training samples, which are drawn through replacement (bagging). The decision trees are then combined into a ‘forest’ to predict the final output, performing better than individual decision trees. Unlike linear models, RF can capture complex non-linear relationships between predictors and the target variable.

**Figure 8:**
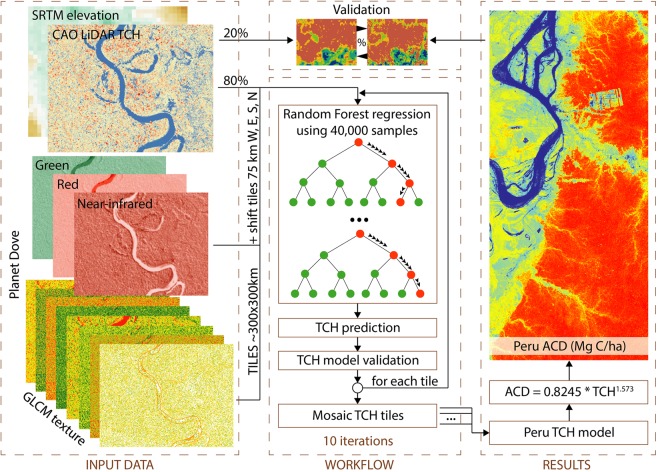
Methodological workflow for aboveground carbon density (ACD) estimation.

We used 12 predictors in the RF regression to estimate TCH, namely green, red and near-infrared bands, the eight GLCM textures and SRTM elevation (Fig. 8, Supplementary Fig. 1). We did not include the blue band since it’s the most sensitive to atmosphere and might introduce unwanted artifacts in the analysis. After being co-aligned and stacked, the 1-ha resolution layers covering the entire Peru were tiled mostly into 300 × 300 km tiles, with larger tiles towards the land borders or Pacific coast of Peru, where the LiDAR coverage was sparser. These tiles have an overlap of 50% (150 km) on each side, meaning that pixels further away from the borders were predicted 4 times by the RF regression. To account for the subjectivity of tiles location when creating them, each tile was shifted with 25% (75 km) towards West, East, South, and North.

The 6,677,177 ha of LiDAR data were split into 80% (5,341,742 ha) for RF regression training and validation and 20% (1,335,435 ha) were kept for the final validation of the country-wide TCH estimation and were not used in any of the RF workflow (Fig. 8). From here onwards, we call a local RF model a model that was applied for one tile of approximate 300 × 300 km. For each local RF model, we used 40,000 samples of 1-ha LiDAR TCH values for training the model, while the remaining samples were used to validate the local model. Two main parameters need to be set up in an RF regression approach, namely the number of trees to grow the forest (*Ntree*) and the number of variables randomly sampled as candidates for each split (*mtry*)^59^. For the latter, we used 4 variables to be considered at each split (*p*/3, where *p* is the number of predictors), while growing 250 trees. This number of trees were decided after running multiple country-wide tests that showed no significant improvement in error rate by increasing the *Ntree* beyond 250 trees. This lower value also ensured a faster computation for the RF regression.

After training and validating more than 200 local RF models (Supplementary Fig. 2), five country-wide models of TCH estimates were produced for each case of tiles positions (unshifted, shifted W, E, S, N) (Supplementary Fig. 3). To minimize the discontinuities between the tiles, a mosaicking procedure based on blending the pixel values of the overlapping areas was used. The blend values are the result of a weighting procedure that consider the distance from the pixel to the edge of the tile, within the overlapping area. The five TCH models were then combined into a single TCH mosaic by averaging the overlapping pixel values. To ensure the robustness of our RF approach, the workflow was repeated 10 times and these models too were averaged to obtain the final Peru-wide TCH estimation (Fig. 8).

### Estimating ACD and associated uncertainties

Converting the estimated TCH to ACD was done using the calibration equation proposed by Asner *et al*.^8^ (Eq. (1)). This equation was developed using a permanent inventory plot network located in diverse ecosystems from which the ACD was derived using allometric equations. The calibration between LiDAR-derived TCH and field-measured ACD resulted in a mean error of 11.6%, in conditions of extreme heterogeneity of biological and land use diversity in the plot network^8^. In the end, our Peru-wide maps of TCH and ACD were validated against the remaining 20% validation samples, from which performance statistics were extracted.

1 $${\rm{ACD}}=0.8245\times {{\rm{TCH}}}^{1.573}$$

We computed uncertainties for TCH and ACD estimation independently. In case of TCH, we grouped the RMSE results into 10 bins using natural breaks method and fitted a function to obtain how the errors are changing in relation to the estimated TCH, both in meters and percentage of the estimated TCH. In the case of ACD, we used the same method to fit a function to model the estimation error in terms of both absolute (Mg C ha^−1^) and percentage values of estimated ACD. For ACD, we combined this error with the second type of error that resulted after calibrating the LiDAR-measured TCH to field-measured ACD by Asner *et al*.^8^ by computing the square root of the sum of the two squared errors (Eq. (2)).

2 $${u}_{c}(ACD)=\sqrt{u{(AC{D}_{field})}^{2}+u{(AC{D}_{RFestimated})}^{2}}$$

where *u*_*c*_(*ACD*) is the combined uncertainty for ACD, *u*(*ACD*_*field*_) is the uncertainty of calibrating LiDAR-measured TCH to field-measured ACD, and *u*(*ACD*_*RFestimated*_) is the uncertainty of our estimated ACD using the RF approach.

## Supplementary information

Supplementary Information

## Data Availability

The data that support the findings of this study are available from Planet Labs Inc. and Global Airborne Observatory (GAO), but restrictions apply to the availability of these data, which were used under license for the current study, and so are not publicly available. GAO data is available upon request from G.P.A.
