# Supplementary material for: Monitoring tropical forest carbon stocks and emissions using Planet satellite data
Source: Sci Rep. 2019 Nov 28;9:17831. doi: 10.1038/s41598-019-54386-6 (PMC6882785; doi:10.1038/s41598-019-54386-6)
Supplement: Supplementary file 1 — Supplementary Information [file 41598_2019_54386_MOESM1_ESM.pdf]

Monitoring tropical forest carbon stocks and emissions using Planet satellite data

Ovidiu Csillik[1,*], **Pramukta Kumar**[1], **Joseph Mascaro**[2], **Tara O'Shea**[2], **Gregory P. Asner**[1]

[1] Center for Global Discovery and Conservation Science, Arizona State University, Tempe, AZ

[2] Planet Labs Inc., San Francisco, CA

Supplementary Information

Computational environment and data preparation

We used a high-performance computing cluster with a theoretical CPU performance of 108 Teraflops and made of computing nodes of Dell PowerEdge R430 with Intel Xeon E5-2680 2.5 GHz processors (24 cores), 1.6 TB SSD local storage and 8 × 16 GB 2133 MT/s registered dual in-line memory module (RDIMM). We developed, tested and apply our codes using R programming language[1].

LiDAR TCH, Planet Dove spectral, GLCM textures and SRTM elevation were resampled at 1-ha resolution, co-aligned to make sure the 1-ha pixels overlap perfectly and projected into WGS 84/UTM Zone 18S. To deal with the highly demanding computation of GLCM textures, we created tiles of 10 × 10 km with overlap, that were in the end stitched and resampled to 1-ha. The 'glcm' R package[2] was used to compute GLCM features. Other R packages used for computations include, but not limited to, 'raster'[3], 'rgdal'[4], and 'randomForest'[5].

Planet imagery are commercial, and the costs vary according to many factors, like the area of interest, processing level or product type. The costs of airborne LiDAR data were approximately $0.01 per hectare over the entire Peru, much cheaper than traditional field-plot inventory approaches[6]. We used free institutional high-performance computing, but users interested can also take advantage of other free computational resources, like Google Earth Engine[7].

Variables importance in RF regressions

One important characteristic of an RF regression is that it gives information of variables importance, i.e. which variable had the most predictive power in estimating TCH. A robust and informative measure is the percentage increase in mean squared error (% IncMSE), which calculates the increase in MSE of predictions when a variable is being permuted (with values randomly shuffled). In our Peru-wide TCH estimations, we averaged these variables over multiple runs and resulted that the SRTM elevation had the highest importance (median 149 % IncMSE), followed by the near-infrared band (median 100) (Figure S3). Red band (median 46), green band (median 31) and GLCM textures derived from the green band (medians between 28 and 51) had lower % IncMSE due to their correlation. From the GLCM textures, Correlation (median 51 % IncMSE), Contrast (median 37) and Second Moment (median 36) had the highest predictive power (Figure S3). These measures seemed to be more sensitive to vegetation structure, with Correlation measuring the occurrence probability of pixel pair values (similar to autocorrelation), Contrast measuring local grey level variation, and Second Moment calculating the uniformity of the gray level distribution within the image.

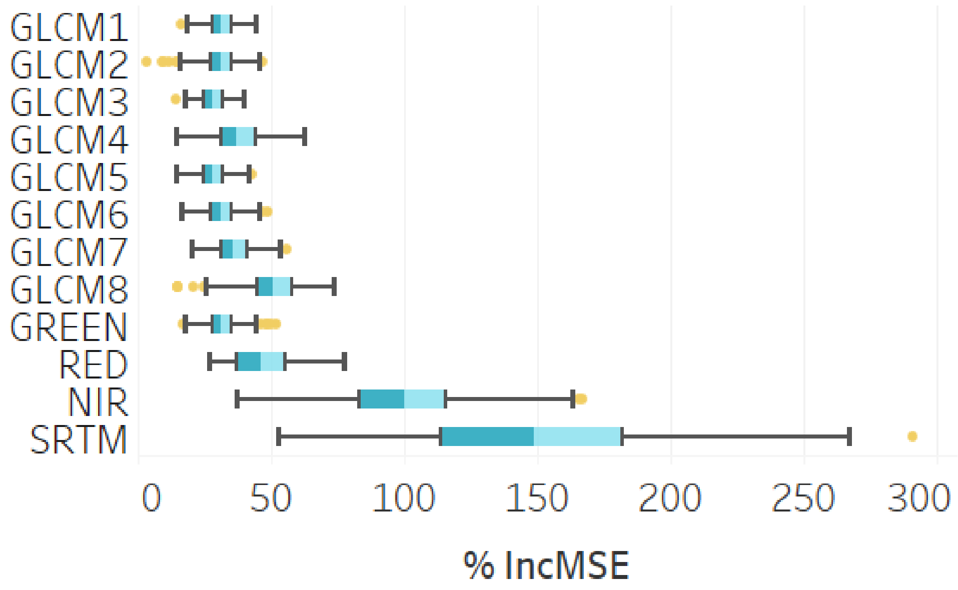

Figure S1. Mean Decrease Accuracy (% IncMSE) resulted from aggregating the results from all RF local models used for the Peru-wide TCH estimation. GLCM 1 to 8 stand for GLCM Mean (1), Variance (2), Homogeneity (3), Contrast (4), Dissimilarity (5), Entropy (6), Second Moment (7), and Correlation (8). Green, red, near-infrared band, and SRTM elevation layers complete the list of RF variables used.

Local Random Forest regression models for TCH estimation

Applying local RF models across the Peru resulted in variable results in terms of accuracy of TCH estimations for each tile, in accordance to a number of factors, like the heterogeneity of ecosystems overlapping the tile, altitudinal range, LiDAR coverage or vegetation structure (Figure S1). TCH estimates from individual tiles and their shifting (W, E, S, N) showed that RF results are sensitive to the tile location and its characteristics (Figure S2). However, the 5 country-wide models constructed from combining these tiles showed relative agreement and less variability (Figure S2). Note that 5 country-wide models composed one of the 10 Peru-wide models that were aggregated to obtain the final model. Aggregating thousands of RF models ensured the robustness of our RF approach by mitigating possible inconsistencies of one or more local RF models.

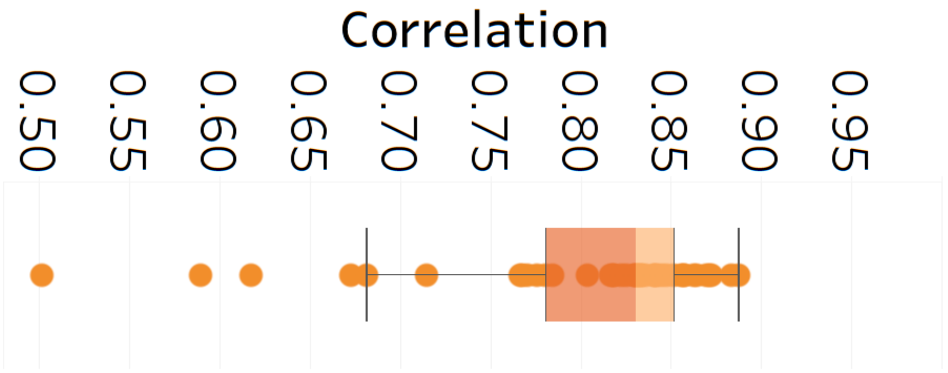

Figure S2. Boxplot of correlation values for the main tiles used in this study, between RF-estimated TCH and LiDAR-derived TCH. The mean correlation was 0.80, within a range of 0.50-0.88 and standard deviation of 0.08.

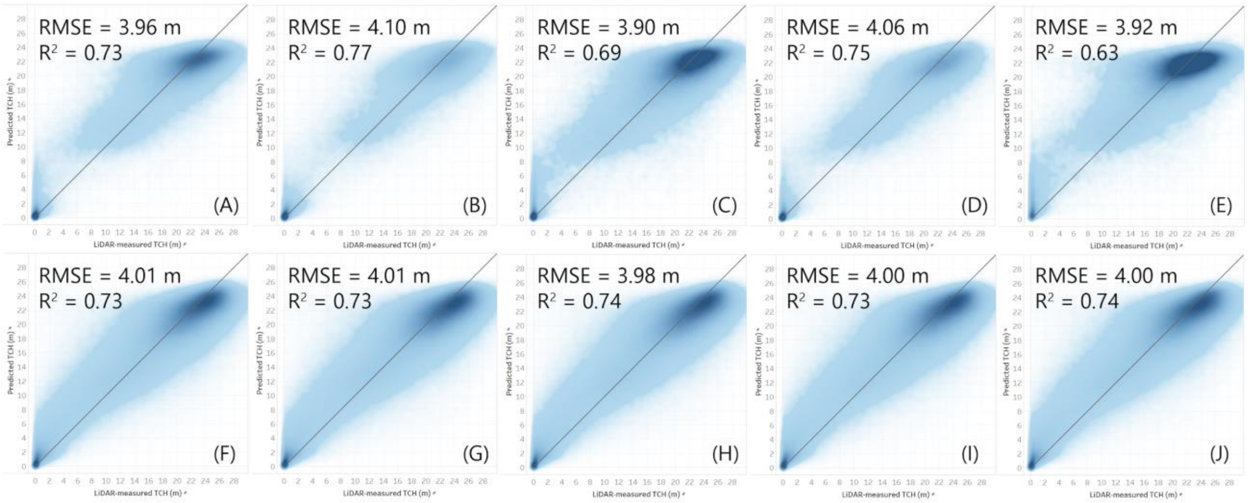

Figure S3. RF results for TCH estimated for a tile and its shifts to W, E, S, and N (a-e) and for the corresponding country-wide RF results (f-j), which together form one of the 10 national TCH estimates. While there was variability between tiles and their shifting, aggregating results to country-wide models reduced the variability of TCH estimations and increased the robustness of the proposed RF workflow (f-j). Note that the horizontal axis shows LiDAR-measured TCH and vertical axis the RF-estimated TCH.

References

1. Team, R Core. R: A language and environment for statistical computing. (2018).

2. Zvoleff, A. Package ‘glcm’. (2016).

3. Hijmans, R. J. *raster: Geographic Data Analysis and Modeling*. (2017).

4. Bivand, R., Keitt, T. & Rowlingson, B. *rgdal: Bindings for the ‘Geospatial’ Data Abstraction Library*. (2018).

5. Liaw, A., Wiener, M. & Others. Classification and regression by randomForest. *R news* **2**, 18–22 (2002).

6. Asner, G. P. *et al.* Targeted carbon conservation at national scales with high-resolution monitoring. *Proceedings of the National Academy of Sciences* **111**, E5016–E5022 (2014).

7. Gorelick, N. *et al.* Google Earth Engine: Planetary-scale geospatial analysis for everyone. *Remote Sens. Environ.* **202**, 18–27 (2017).
